# The response of rumen pH, fermentation parameters and rumen bacteria to feeds of different concentrate to roughage ratios in buffalos

**DOI:** 10.3389/frmbi.2022.1053794

**Published:** 2023-01-09

**Authors:** Rongjiao Wang, Shichun He, Dan Huang, Dongwang Wu, Hongen Peng, Shaoying He, Taiqing Guo, Tao Chen, Xianhai Fu, Changguo Chen, Latie Jiaka, Min He, Dingzhou Song, Xiujun Huang, Huaming Mao, Qing Li

**Affiliations:** ^1^ Panzhihua Academy of Agricultural and Forestry Sciences, Institute of Animal and Veterinary Medicine, Panzhihua, China; ^2^ Yunnan Provincial Key Laboratory of Animal Nutrition and Feed Science, Faculty of Animal Science and Technology, Yunnan Agricultural University, Kunming, China; ^3^ Animal Husbandry station in Mangshi, Dehong Prefecture, Mangshi, Yunnan, China

**Keywords:** buffaloes, roughage, digestibility, ruminal fermentation parameters, ruminal bacteria

## Abstract

This experiment was conducted to study the regularity influence in apparent digestibility, rumen fermentation parameters, and relative bacterial abundance in buffaloes with different concentrate to roughage ratios. Three adult female barren buffaloes with permanent rumen fistulas were fed five diets with concentrate to roughage ratios of 20:80, 35:65, 50:50, 65:35, and 80:20 according to an incomplete Latin square design of 3×5, respectively. The pre-feeding period of each period was 12 d. From day 13 to 15 of the experiment, the feed intake of each buffalo was accurately recorded and all feces were collected. Samples of diet and feces were collected for nutrient analysis. Rumen fluid was collected to determine rumen fermentation parameters, and rumen bacteria were analyzed by 16S rRNA sequencing. After 2 hours of feeding in the morning on the 15th day of the experiment, rumen bacteria were analyzed. The daily gain and DMI (G/kgW0.75) reached the highest at the concentrate to roughage ratio of 50:50, and the apparent digestibility of ADF NDF NDICP ADICP reached the highest at the concentrate to roughage ratio of 35:65. The weight loss of the experimental animals when the concentrate to roughage was 80:20. Rumen pH decreased with the increase of concentrate level, starch content and NFC content. The rumen ammonia nitrogen concentration increased with the increase in the concentrate to roughage ratio and protein content. The concentration of total volatile fatty acids (mmol/L) in the rumen decreased with the increase of NDF but increased with the increase of NFC and CP, and the acetic acid proportion increased with the increase of NDF. There were differences in the relative abundance of rumen bacterial microbiota with the different concentrate to roughage ratios. Moreover, the relative abundance of each bacterial microbiota changed regularly with the change of concentrate to roughage ratio. The relative abundance of *Bacteroidetes*, *Firmicutes*, and other rumen core microbiota varied linearly or nonlinearly with the change of concentrate to roughage ratio. This suggests that the relative abundance of the ratio of rumen microbiota can be used to specify or measure rumen health and subacute rumen acidosis/rumen acidosis.

## 1 Introduction

Buffalo is a large herbivore endemic to tropical or subtropical regions. Buffalo is not only resistant to rough feeding, but also has the characteristics of strong adaptability, high temperature, and humidity resistance, and low feeding cost. Buffalo made an important contribution to farming in developing countries (Wanapat et al., 2009). Among ruminant breeds, buffalo has a stronger fiber digestion capacity than other cattle breeds, and some researchers have noted this. Sirohi et al. (2013) found that the copy number of buffaloes’ *Filamentous succinate* and *Ruminococcus Albicans* was significantly higher than that of other cattle. The rumen microbes were distinct when different concentrate to roughage ratios of diet, which was further found in buffalo. Wanapat et al. (Wanapat and Cherdthong, 2009) fed buffaloes with concentrate to roughage ratios of 0:10, 2.5:7.5, 5:5, and 7.5:2.5. In the experiment, it was found that *succinic acid bacilli* were the most abundant in buffalo rumen fiber-degrading bacteria, followed by *Ruminococcus xanthococcus* and *Ruminococcus albus*.

The relative abundance of *Prevorella*, *Treponema*, and *Mycoplasma anaerobe* decreased, while the relative abundance of *Ruminococcus* and *Clostridium* increased in the rumen after the buffalo was fed a high concentration level diet (Mao et al., 2013). Joshi et al. used 16S rRNA sequencing to study the rumen bacterial diversity of buffaloes under three concentrate to roughage ratios (5:5, 2.5:7.5, and 0:10). The results showed that the quantity of *Ruminococcus* and *Fibrobacter* in the 100% roughage group was significantly higher than that in the other two groups. *Prevotella* was the most abundant in the 50% roughage group (Pitta et al., 2013). Nevertheless, it is not enough to explain the change rule of rumen microbes when the concentrate to roughage ratios size range is not large enough.

Based on the results of previous studies, this study hypothesized that there is a regularity influence in rumen fermentation parameters and a relative abundance of rumen bacteria under different concentrate to roughage ratios. Therefore, the primary objective of this study was to explore whether this linear or nonlinear regularity influence exists. If so, what are the specific rules?

## 2 Materials and methods

### 2.1 Experimental animals and experimental design

Three ruminal fistulated buffaloes (average body weight: 365 ± 25 kg. 25 months old) and 5 diets were used in a 3×5 Latin square design. Each diet passed three feeding tests. All buffaloes were allocated to 3 pens.

The diets and clean water were available throughout the entire experiment. The ratio of concentrate to roughage was 20:80, 35:65, 50:50, 65:35, and 80:20, respectively. The roughage consists of 1/3 oat hay, 1/3 whole corn silage, and 1/3 dry rice straw.

### 2.2 Sample collection

The diets in each treatment were weighed and recorded daily to calculate individual dry matter intake (DMI). Diet samples of approximately 500g from each treatment were collected, dried at 65 °CC, ground through a 60 mesh sieve, and stored in a valve bag for nutritional analysis. To measure animal daily gain, buffalos were weighed before feeding using an electronic scale at the beginning and end of the formal experiment. Feces were collected, mixed, and weighed daily during a 3-day formal trial period. Approximately 2 kg samples of feces were dried at 65°C for 48 h. Total tract digestibility was calculated based on the differences in nutrient concentration between feces and diets. On 15 d, Ruminal fluid samples were collected 2 h after the morning feeding from rumen-fistulated.

### 2.3 Chemical analysis

Samples of roughage grass, hay refused, and feces were ground in a mill to pass a 1-mm screen and analyzed for ether dry matter (DM), Organic matter (OM), neutral detergent fiber (NDF), acid detergent fiber (ADF), crude ash (Ash), crude protein (CP), non-fibrous carbohydrate (NFC), acid detergent lignin (ADL), ether extract (EE), calcium (Ca), phosphorus (P), neutral detergent insoluble crude protein (NDICP), acid detergent insoluble crude protein (ADICP) (Al-Suwaiegh et al., 2002; Zhao et al., 2015).

Rumen fluid PH was measured in time by PHS-3C pH meter after rumen fluid collection. The acidity meter was preheated and calibrated half an hour before the formal determination.

NH_3_-N was determined by the phenol-hypochlorous acid colorimetric method. Rumen fluid samples were filtered through 4 layers of gauze. The 6 mL sample was placed in a 10 mL centrifuge tube. 40 μL of the supernatant was centrifuged at 12000×g for 20 min. 2.5mL phenol chromogenic agent and 2.0mL sodium hypochlorite reagent were added to the supernatant, respectively. After that, the supernatant was thoroughly mixed by shaking and placed in a 37 °C water bath for 30 min. Colorimetric analysis of the supernatant was performed using a visible spectrophotometer at a wavelength of 550 nm. Standard curves were drawn by NH_4_Cl standard solution (y = 0.0423x+0.0157, R^2 =^ 0.9985). NH_3_-N concentration was calculated based on colorimetric results and standard curves.

VFA was determined by gas chromatography-mass spectrometry. Sample pretreatment: A sample of 20 μL rumen fluid was placed in a 2 mL EP tube, then 380 μL ddH2O was added and vortexed for 30 s. 200 μL of diluted sample was added with 100 μL 15% phosphoric acid, 20 μL of 75 μg/mL internal standard (isohexanoic acid) solution and 280 μL of diethyl ether homogenate for 1 min, and centrifuged at 12000 rpm for 10 min at 4°C. Finally, the supernatant was taken and tested on the machine.

Chromatographic determination conditions: Agilent HP-INNOWAX Capillary column (30 m*0.25 mm ID*0.25 μm). The injection volume was 1 μL, and the split ratio was 10:1. The inlet temperature was 250 °C; the Ion source temperature was 230 °C. The initial temperature of program heating is 90 °C, then the temperature was raised to 120 °C at 10 °C/min. Then the temperature increased by 150 °C at 5 °C/min. Finally, the temperature was raised to 250 °C for 2 min at 25 °C/min. The carrier gas was helium with a flow rate of 1.0 mL/min.

### 2.4 Amplification and sequencing

DNA extraction: Cell lysis of rumen fluid was achieved by beading in the presence of 4% (w/v) sodium dodecyl sulfate (SDS), 500 mM NaCl, and 50 m EDTA. The buffer acts to protect the released DNA from degradation by DNase, which is very active in rumen fluid samples. After beading, impurities and SDS were removed by ammonium acetate precipitation, and nucleic acid was removed by isopropanol precipitation. Zymo Research BIOMICS DNA Microprep Kit was used for sample gDNA purification (Wanapat and Cherdthong, 2009; Wallace et al., 2019).

Additive sequencing with Illumina sequencing technology was used in the experiment. The 16S rRNA of prokaryotes, the ITS gene of fungi, or specific functional genes can be used in taxonomic identification (Kuczynski et al., 2011). The following universal primers were applied for the amplification of the V4 region of the 16S rRNA gene. Primer5’-3’: 515F (5’-GTGYCAGCMGCCGCGGTAA-3’) and 806R(5’-GGACTACHVGGGTWTCTAAT-3’). The conditions of the real-time PCR assays were as follows: for 16S rRNA: 1min at 94°C for initial denaturation (1 cycle), 20 s at 94°C for denaturation, 30 s at 54°C for annealing and 30 s at 72°C for extension (30 cycles), and a final extension period of 10 min at 72°C. Three separate PCRs for each sample were pooled for processing.

Libraries were constructed using NEBNext Ultra II DNA Library Prep Kit for Illumina from NEW ENGLAND BioLabs. PE250 was used for high-throughput sequencing, and the sequencing Kit was Illumina Hiseq Rapid SBS Kit v2(FC-402-4023 500 Cycle).

### 2.5 Data analysis

Apparent digestibility (Ekelund et al., 2006):


Apparent digestibility = (nutrient intake - nutrient excretion)/nutrient intake ×100%.

The original offline data obtained by sequencing were spliced and filtered to obtain high-quality target sequences for subsequent analysis. Subsequent operation of bioinformatics research (http://www.drive5.com/usearch) and QIIME completed, such as statistics and drawing mainly using R, Python, and Java (Caporaso et al., 2010; Dalgaard and R. Development Core Team, 2010).

Data quality control: Splice a two-ended sequence using FLASH. Each sample sequence was isolated from raw reads based on Barcode, and the Barcode sequence was truncated. Then use QIIME for quality control. Filter out sequences with an average mass of less than 25. The sequence length of less than 200bp was removed. The sequence with more than 2 fuzzy bases (N) was removed. The Uchime algorithm and gold database were used to remove chimeras, and effective data Effiective Tag was obtained.

OUT clustering, species annotation, and evolutionary tree construction: Based on Research (http://drive.com/uparse/) software. The UPARSE algorithm was used for OTU clustering at the consistency level of 97%, and the sequence with the highest frequency in each OTU was selected as the representative sequence of OUT. UCLUST classification and the SILVA database were used for annotation analysis. Multiple alignments of representative sequences are performed using PyNAST. Use FastTree to build an evolutionary tree. All samples are homogenized, and the sample with the least amount of data is taken as the standard for resampling.

Community composition analysis: R language was used for various data conversion, ggplot2 package mapping.

Alpha diversity analysis: R language was used for analysis. PD index was calculated using the package Picante, and other indexes were calculated using the package Vegan. The Wilcoxon rank sum test uses the Wilcox. test function of the stats package, and the Kruskal. the test function is used for the Kruaksl-Wallis rank sum test of the two groups. Multiple comparisons use the package agricolae.

Beta diversity analysis: R language was used for analysis. Calculate the Unifrac distance using the package GuniFrac, and calculate the Bray-Curtis and Jaccard distances using the Vegan package’s vegdits function. PCoA analysis uses a package ape. PCA and NMDS analyses were performed using the vegan package. Cluster analysis uses the hclust function of the package stas. Anosim and PerMANOVA are calculated using the vegan’s anosim and adonis functions, respectively.

Difference species analysis: LefSe analysis using LefSe tools (https://bitbucket.org/biobakery/biobakery/wiki/Home). randomForest package using R language is used for random forest analysis. Metastas analysis using R language script, calculation steps (https://journals.plos.org/ploscompbiol/article?id=10.1371/journal.pcbi.1000352) for reference.

Community function prediction: Cluster and annotate the sequencing data of the 16S rRNA gene based on the SILVA database, and linearly transform the calculated results based on the pre-calculated correlation matrix to obtain the microbial classification spectrum based on the KEGG database. The results were corrected according to the copy number of the 16S rRNA gene in the genome of different bacteria in NCBI. The classification information was linearly predicted based on the functional gene profiles of microorganisms in the KEGG database.

Excel was used to organize the test data, and SPSS 22.0 and Origin were used to conduct variance analysis, unary linear regression analysis, correlation analysis between variables, curve regression analysis, and Duncan’s multiple comparison test. Some software and database version information: QIIME v1.9.0, Usearch 10.0.240, R language: 3.6.0, Python: 3.7.4, SILVA database: 132. The results were expressed as mean ± standard deviation. *P*<0.05 was used as the difference significance criterion, and *P*<0.01 was used as the criterion of extremely significant difference. Sequencing was performed by PanoMIX Metabolic Biotechnology LTD. Sequence Read Archive PRJNA888478. Records will be accessible with the following link after the indicated release date: https://www.ncbi.nlm.nih.gov/biosample/31218934, https://www.ncbi.nlm.nih.gov/biosample/31218935,https://www.ncbi.nlm.nih.gov/biosample/31218936, https://www.ncbi.nlm.nih.gov/biosample/31218937 , https://www.ncbi.nlm.nih.gov/biosample/31218938, https://www.ncbi.nlm.nih.gov/biosample/31218939, https://www.ncbi.nlm.nih.gov/biosample/31218940, https://www.ncbi.nlm.nih.gov/biosample/31218941, https://www.ncbi.nlm.nih.gov/biosample/31218942, https://www.ncbi.nlm.nih.gov/biosample/31218943, https://www.ncbi.nlm.nih.gov/biosample/31218944, https://www.ncbi.nlm.nih.gov/biosample/31218945, https://www.ncbi.nlm.nih.gov/biosample/31218946, https://www.ncbi.nlm.nih.gov/biosample/31218947, https://www.ncbi.nlm.nih.gov/biosample/31218948.

## 3 Results

### 3.1 Dietary nutrients

As can be seen from **Table 1**, the contents of Ash, NDF, ADF, ADL, and NDFn in diets decreased linearly, while other nutrients increased linearly with the increase of concentrate to roughage ratio.

**Table 1:** Comparison of nutrient composition of experimental groups with different concentrate to roughage ratios (dry matter basis).

Project	20:80	35:65	50:50	65:35	80:20
DM	48.1±0.02^eE^	53.26±1.97^dD^	58.09±0.00^cC^	65.53±1.38^bB^	73.7±0.98^aA^
OM	91.02±0.01	91.21±0.13	91.58±0.00	91.83±0.01	92.12±0.01
CP	9.69±0.00^eE^	12.15±0.29^dD^	14.89±0.00^cC^	17.52±0.09^bB^	20.07±0.04^aA^
EE	1.95±0.00	2.01±0.03	2.1±0.00	2.17±0.01	2.26±0.01
Ash	8.98±0.06	8.79±0.12	8.42±0.00	8.17±0.46	7.88±0.02
Ca	0.6±0.00^eE^	0.72±0.01^dD^	0.85±0.00^cC^	0.98±0.01^bB^	1.1±0.01^aA^
P	0.23±0.00^eE^	0.28±0.01^dD^	0.33±0.00^cC^	0.38±0.01^bB^	0.43±0.00^aA^
NDF	51.71±0.02^aA^	44.84±0.75^bB^	36.9233±0.01^cC^	29.5±0.15^dD^	22.21±0.07^eE^
ADF	35.67±0.02^aA^	30.65±0.54^bB^	24.83±0.01^cC^	19.39±0.11^dD^	14.04±0.06^eE^
ADL	4.13±0.01^aA^	3.53±0.07^bB^	2.86±0.00^bcBC^	2.23±0.02^cdCD^	1.61±0.02^dD^
NDICP	1.44±0.01^cC^	1.6±0.03^bB^	1.76±0.00^abAB^	1.93±0.01^abAB^	2.09±0.01^aA^
ADICP	0.78±0.00	0.8±0.01	0.82±0.00	0.84±0.00	0.86±0.00
NFC	29.11±0.01^eE^	33.81±0.52^dD^	39.43±0.01^cC^	44.57±0.05^bB^	49.68±0.03^aA^
NDFn	50.27±0.02^eE^	43.25±0.76^dD^	35.16±0.01^cC^	27.57±0.17^bB^	20.12±0.08^aA^
STARCH	14.76±0.99^eE^	20.41±0.80^dD^	26.06±0.60^cC^	31.71±0.41^bB^	37.36±0.22^aA^
GE cal/g	3735.97±21.4	3765.74±37.45	3795.52±53.5	3825.29±69.55	3855.06±85.6

Different lowercase letters in the same line indicate P<0.05, and both capitals are different for P<0.01.

### 3.2 Dietary intake and daily gain

As can be seen from **Table 2**. The total feed intake, DMI (g/kg W0.75), daily gains a general trend of increasing first and then decreasing with the ratio of concentrate to roughage increasing. With the increase of concentrate to roughage, the roughage intake showed a decreasing trend.

**Table 2:** Feed intake and daily gain of experimental groups with different concentrate to roughage ratio (dry matter basis).

Project	20:80	35:65	50:50	65:35	80:20
The total feed intake (kg)	7.31±0.57	8.29±0.46	8.33±0.57	8.18±0.93	6.4±2.31
DMI (g/kg W0.75)	68.10±6.34	78.85±7.01	80.59±4.82	78.05±6.87	59.44±16.27
Daily gain (kg)	1.42±0.34^aAB^	1.22±0.34^aA^	1.98±0.65 ^aA^	1.56±1.43 ^aA^	-0.53±0.87^bB^

Different lowercase letters in the same line indicate P<0.05, and both capitals are different for P<0.01.

The total feed intake and daily gain were highest in the 50:50 group and lowest in the 80:20 group. The maximum daily gain of the 50:50 group was 1.98 kg/d, and the minimum daily gain of the 80:20 group was -0.53 kg/d, which was significantly lower than that of the 35:65, 50:50, and 65:35 groups. Total feed intake and DMI (g/kg W0.75) did not differ significantly between the five groups. The maximum value was in the 50:50 group and the minimum value was in the 80:20 group.

### 3.3 Apparent digestibility of the diet

The Dry matter apparent digestibility (DMD) presented an increasing trend with the rise of the ratio of concentrate to roughage. The DMD of the 80:20 and 65:35 groups were significantly higher than that of the other three groups. The lowest DMD in the 20:80 group was significantly lower than that in other groups.

The apparent digestibility of crude protein (CPD) increased with the increase of the concentrate to roughage ratio. The CPD was higher in 50:50, 65:35, and 80:20 groups, with no significant difference among groups, but significantly higher than in the other two groups. The CPD of the 35:65 group was significantly higher than that of the 20:80 group. The CPD in the 20:80 group was significantly lower than that in other groups.

The highest apparent digestibility of neutral detergent fiber (NDFD) was found in the 35:65 group (64.17%), which was significantly higher than that in other groups. Followed by the 65:35 group, 50:50 group, 80:20 group, and 20:80 group, but there was no significant difference between the four groups.

As can be seen from **Table 3**, DMD and OMD increased with the increase of concentrate to roughage ratio. The maximum values of ADFD, ADICPD, NDICPD, AshD, ADLD, and NDFnD were in the 35:65 groups. The maximum value of CPD and EED appeared in the 50:50 group.

**Table 3:** Apparent digestibility of each nutrient in diets with different ratios of concentrate to roughage.

Apparent digestibility(%)	20:80	35:65	50:50	65:35	80:20
DMD	53.19 ± 3.17^dC^	63.11 ± 2.18^bcABC^	64.32 ± 2.15^bcABC^	73.11 ± 1.35^aAB^	74.78 ± 2.36^aA^
CPD	69.17 ± 5.65^cAB^	71.84 ± 1.68^bAB^	76.10 ± 2.75^aA^	75.50 ± 2.61^aA^	76.06 ± 2.99^aA^
EED	80.68 ± 2.78^aA^	75.62 ± 1.29^aA^	81.66 ± 1.96^aA^	71.91 ± 1.27^abA^	73.05 ± 1.60^abA^
ADFD	39.62 ± 1.32^aA^	47.11 ± 1.95^aA^	41.23 ± 2.10^aA^	43.12 ± 3.38^aA^	38.95 ± 1.60^aA^
NDFD	45.44 ± 1.14^bcBC^	64.17 ± 2.97^aA^	49.15 ± 1.78^bABC^	50.89 ± 2.66^bABC^	46.65 ± 1.14^bcBC^
ADICPD	23.88 ± 3.53^cC^	38.46 ± 1.09^aA^	30.74 ± 4.34^bB^	30.44 ± 2.36^bB^	24.62 ± 3.75^cBC^
NDICPD	34.27 ± 0.93^bABC^	39.38 ± 1.80^abAB^	38.11 ± 0.98^abAB^	33.78 ± 1.22^bBC^	44.96 ± 1.46^aA^
AshD	16.41 ± 3.21^eD^	23.10 ± 1.44^deCD^	19.85 ± 2.06^deCD^	36.19 ± 0.95^abcABC^	30.93 ± 3.53^bcdABCD^
ADLD	7.51 ± 0.37^bB^	16.73 ± 1.51^bAB^	9.97 ± 0.33^bB^	7.93 ± 0.61^bB^	14.51 ± 1.75^bAB^
OMD	56.82 ± 1.01^cBC^	66.97 ± 4.61^bABC^	68.41 ± 1.78^bAB^	76.39 ± 1.54^aA^	78.53 ± 2.19^aA^
NDFnD	45.75 ± 2.42^bcBC^	65.09 ± 4.16^aA^	49.69 ± 1.90^bABC^	52.09 ± 2.93^bABC^	46.83 ± 1.92^bcBC^
CaD	19.74 ± 1.09^cdABCD^	26.56 ± 1.12^bcBC^	12.38 ± 1.92^dC^	27.21 ± 1.51^bcBC^	17.40 ± 1.56^cdBC^
PD	14.65 ± 2.93^dB^	12.46 ± 0.53^dB^	20.00 ± 3.25^bcdAB^	15.39 ± 4.11^cdB^	30.27 ± 2.06^abcAB^
NFCD	69.76 ± 2.51^bcCD^	67.03 ± 2.22^bcCD^	81.38 ± 1.90^abABC^	91.74 ± 3.31^aAB^	93.60 ± 5.45^aA^

Different lowercase letters in the same line indicate P<0.05, and both capitals are different for P<0.01.

The maximum value of CaD was found in the 65:35 group. The maximum values of DMD, OMD, PD, and NFCD appeared in the 80:20 group. The highest digestibility of all nutrients did not appear in the 20:80 group. The apparent digestibility of other nutrients was displayed in the table above.

### 3.4 pH of rumen fluid at different concentrate to roughage ration

**Figure 1:**
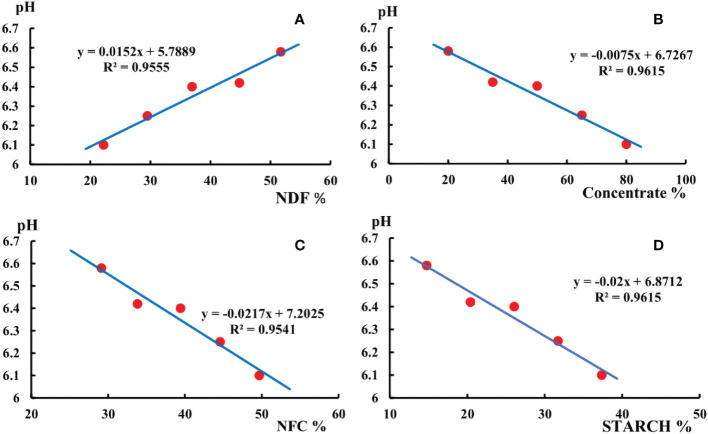
**(A)** shows the pH changed with the content of NDF in the diet. **(B)** shows the pH changed with the concentrate in the diet. **(C)** shows the pH changed with the content of NFC in the diet. **(D)** shows the pH changed with the content of starch in the diet.

From **Figure 1** it can be seen that the rumen fluid pH of all experimental groups was between 6.0 and 7.0, which remained within the range of acidity. The 20:80 group was the highest, 80:20 group was the lowest.

The pH of the rumen fluid decreased as the NFC content, concentrate to roughage ratio, and starch content of the diet increased. The pH of rumen fluid was below 6.4 in both the 65:35 and 80:20 groups. Rumen fluid pH increased with dietary NDF content increasing. The simulation equations are displayed in **Figure 1**.

### 3.5 NH_3_-N of rumen fluid at different ratios of concentrate to roughage

**Figure 2:**
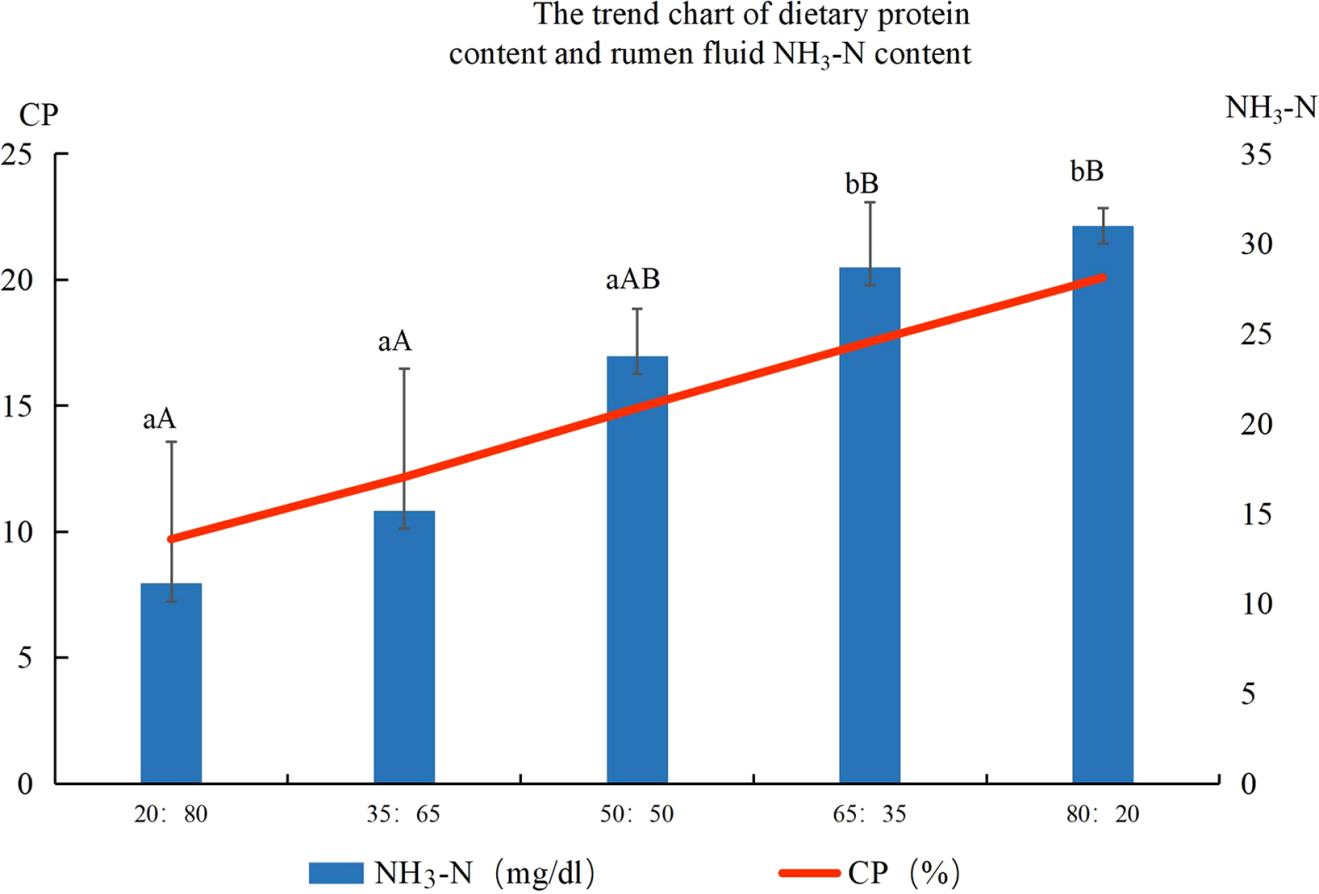
Ruminal fluid ammonia nitrogen concentrations (mg/dl) of different concentrate to roughage ratios Note: Different lowercase letters between groups indicate significant differences, *P*<0.05, capital letters indicate significant differences, *P<0.01*.

NH_3_-N of rumen fluid at different ratios of concentrate to roughage are shown in **Figure 2**. The concentration of NH_3_-N in rumen fluid increased with the increase in dietary protein content. The concentration of NH_3_-N in rumen fluid in the 80:20 group was the highest, which was significantly higher than that in the 20:80 group, 35:65 group, and 50:50 group. The concentration of NH_3_-N in rumen fluid in the 20:80 group was the lowest, which was significantly lower than that in the 80:20 and 65:35 groups. The linear equation NH_3_-N(mg/dl) = 2.0426 (CP%)- 8.4155, R²= 0.9698 was obtained by linear simulation.

### 3.6 Contents of volatile fatty acids in the rumen of different ratios of concentrate to roughage

#### 3.6.1 Contents of volatile fatty acids in the rumen

According to **Table 4**, the concentration of acetic acid is higher than that of propionic acid. The concentrations of total volatile fatty acids, acetic acid, and propionic acid were the highest in group 35:65 and the lowest in group 50:50. The acetic acid concentration in group 35:65 was significantly higher than that in group 50:50.

**Table 4:** Contents of volatile fatty acids in the rumen of different ratios of concentrate to roughage (mmol/L).

Group	Acetic acid	Propionic acid	Isobutyric acid	Butyric acid	Isovaleric acid	Pentanoic acid	Caproic acid	Total volatile fatty acids	Proportion of acetic acid in TVFA
20:80	73.58±5.28^ab^	19.36±2.06	0.76±0.03^dCD^	8.77±1.02^bcBC^	0.58±0.05^dC^	1.2±0.11^b^	0.38±0.05^b^	104.64±8.52	0.70±0.01^bcdAB^
35:65	84.79±14.86^a^	21.15±2.87	0.96±0.09^bcABC^	10.58±1.48^abAB^	0.76±0.07^cC^	1.42±0.17^ab^	0.5±0.05^ab^	120.16±18.38	0.70±0.03^bcdAB^
50:50	67.28±4.27^b^	16.86±0.95	0.87±0.06^cdBCD^	9.98±2.26^bABC^	0.76±0.08^cC^	1.36±0.23^ab^	0.51±0.14^ab^	97.6±7.89	0.69±0.01^cdAB^
65:35	73.42±13.44^ab^	18.46±5.51	1.11±0.14^abAB^	13.26±2.67^aA^	1.07±0.13^bB^	1.58±0.44^ab^	0.59±0.14^a^	109.49±22.28	0.67±0.02^dB^
80:20	76.57±14.03^ab^	20.44±7.85	1.20±0.09^aA^	13.54±4.05^aA^	1.28±0.10^aA^	1.91±0.79^a^	0.58±0.27^a^	115.52±26.39	0.67±0.03^dB^

Different lowercase letters between groups indicate significant differences, P<0.05, capital letters indicate significant differences, P<0.01.

Concentrations of Isobutyric acid, Butyric acid, Isovaleric acid, and Pentanoic acid reach the highest in the 80:20 group and the lowest in the 20:80 group, with significant differences between them. The concentration of Caproic acid was the highest in the 65:35 group and the lowest in the 20:80 group, and the difference between them was significant. Other volatile fatty acid changes were displayed in **Table 4**.

The decreasing trend of acetic acid concentration was not obvious with the increase in concentrate to roughage ratios. However, the proportion of acetic acid in TVFA decreased with the increase in concentrate to roughage ratios. With the increase of dietary NFC content and the decrease of NDF content, the proportion of acetic acid in TVFA showed a decreasing trend, which could be seen in combination with dietary nutritional data.

#### 3.6.2 The proportion of acetic acid in total volatility under different concentrate to roughage ratio diets

**Figure 3:**
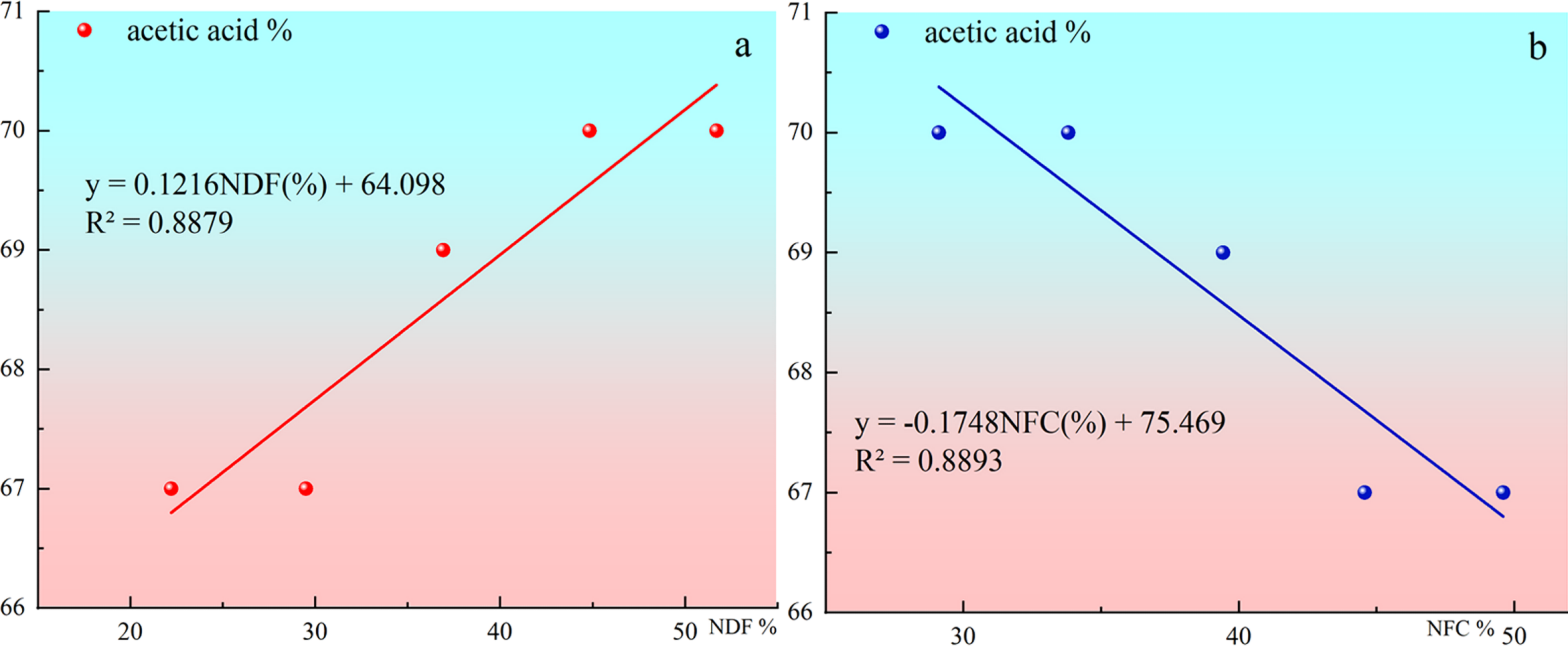
**(A)** shows the Change of acetic acid proportion in total volatile fatty acids with NDF contents in diets. **(B)** shows the Change of acetic acid proportion in total volatile fatty acids with NFC contents in diets.


The change of acetic acid proportion in total volatile fatty acids with NDF and NFC contents in diets is shown in **Figure 3**. Acetic acid concentration did not completely decrease with the increase of concentrate proportion in this study. However, the proportion of acetic acid decreased with the increase of concentrate, which was reflected in that the proportion of acetic acid in total volatile fatty acids gradually increased with the increase of dietary NDF content. The proportion of acetic acid in total volatile fatty acids gradually decreased with the increase of dietary NFC content.

$$\text{Acetic acid}\%\;=\;0.0012\times \text{NDF }\left(\%\right)\;+\;0.641,{\text{ R}}^{2}\;=\;0.8880$$

$$\text{Acetic acid}\%\;=-0.0017\times \text{NFC }\left(\%\right)\;+\;0.7545,{\text{ R}}^{2}\;=\;0.8886$$

### 3.7 Analysis of rumen bacterial alpha diversity

**Figure 4:**
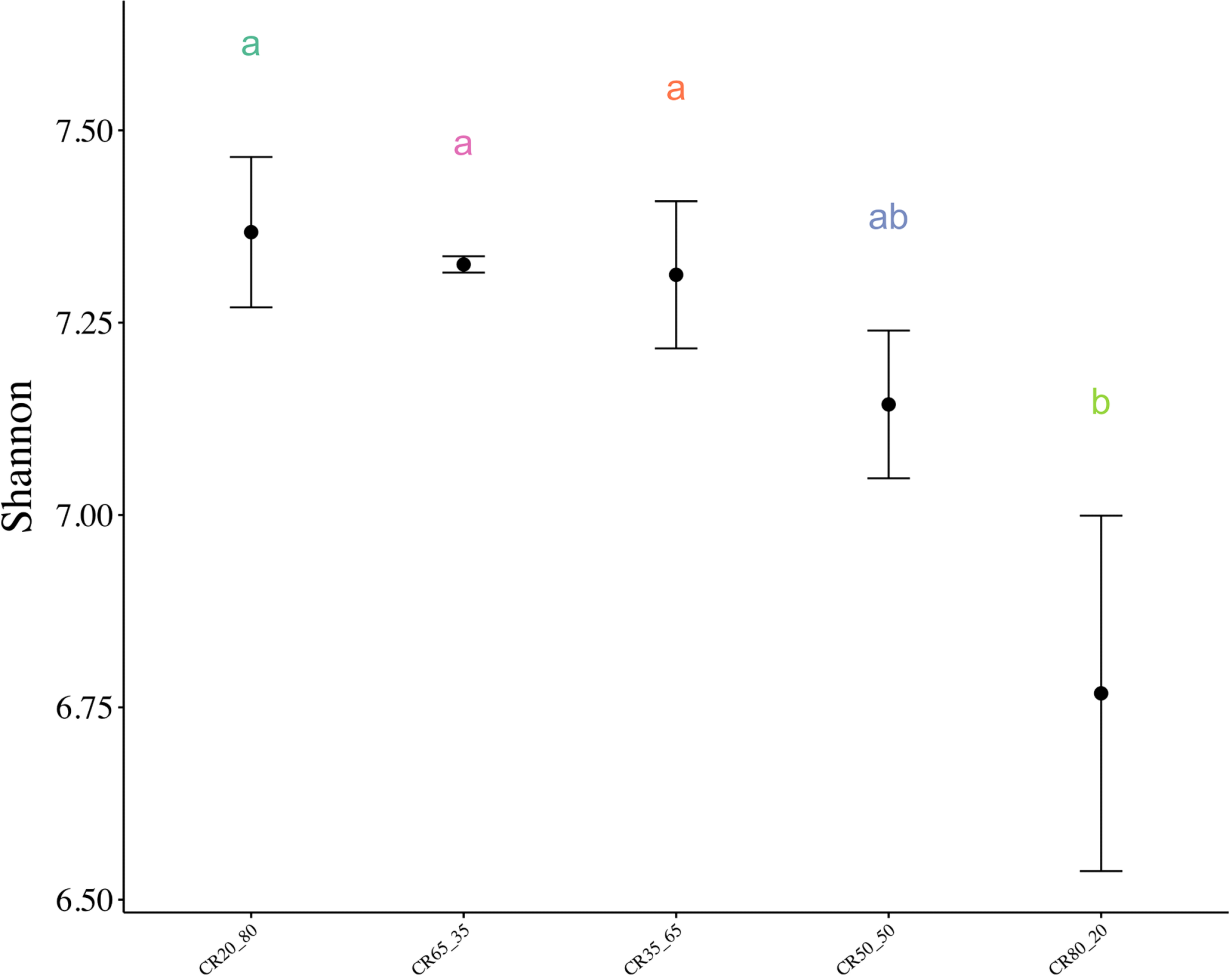
Shannon index of rumen microorganisms with different concentrate to roughage ratios. P < 0.05. Different lowercase letters between groups indicate significant differences, P<0.05.

**Figure 4** shows that the Shannon index of the 80:20 group was significantly lower than that of the other four groups. The Shannon index decreased with the increase of concentrate to roughage ratio, but there were no significant differences among 20:80, 35:65, 50:50, and 65:35. The results suggested that the diversity of rumen fluid in group 80:20 was significantly lower than that in the other 4 groups.

### 3.8 Relative abundance of phylum level

**Figure 5:**
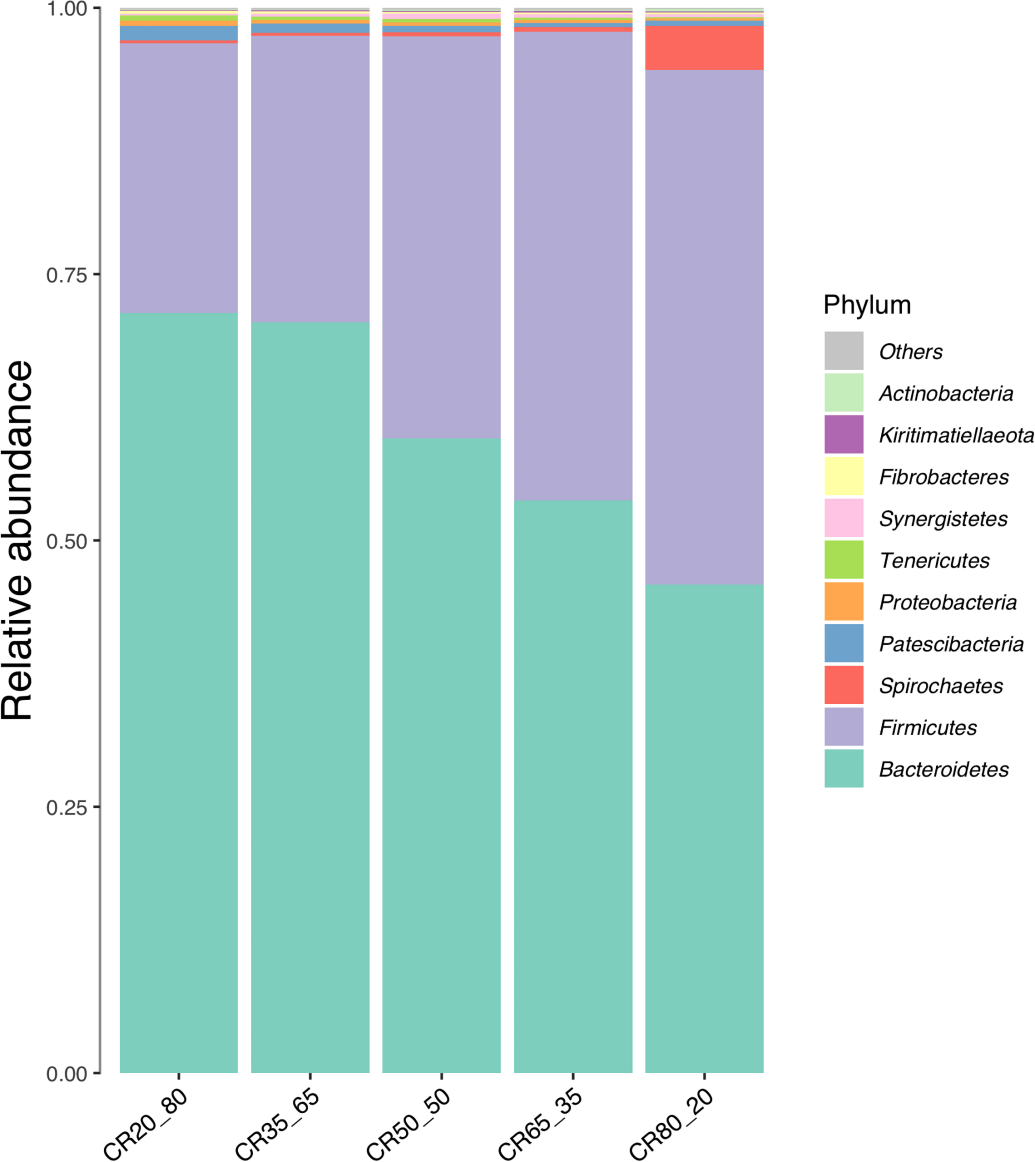
Composition of relative abundance of phylum level in rumen fluid under different concentrate to roughage ratios.

**Figure 5** is a bar chart of the relative abundance of species, showing the relative abundance of the top 10 phyla in the five experimental groups.

Only *Bacteroidetes* and *Firmicutes* had a relative abundance of more than 3%. The relative abundance of the *Bacteroides* was higher than that of the *Firmicutes* in the groups tested, but in the 80:20 group, the *Firmicutes* were higher than the *Bacteroides*. The relative abundance of *Firmicutes* increased and that of *Bacteroidetes* decreased with the increase of concentrate to roughage ratio. In this study, the relative abundance of *Bacteroidetes* decreased with the decrease in pH, which was not difficult to find in combination with the rumen pH data.

Candidate phyla radiation group (CPR) also known as *Patescibacteria* is a special group of bacteria. CPR bacteria were first defined by Brown in 2015 because their cell morphology is an important subset of the smallest bacterial family known to exist on earth (Brown et al., 2015). In this study, the bacterial group was found in the rumen fluid of De Hong’s three-way hybrid buffalo. The overall trend is to decrease with the increase in the ratio of concentrate to roughage.

The relative abundance of spirochaetes ranged from 0.24% to 4.35%. The highest relative abundance was found in the 80:20 group, up to 4.35%. *Proteobacteria*, *kiritimatiellaeota*, *Lentisphaerae synergistetes*, and *fibrobacteres* were all less than 1% in relative abundance.

### 3.9 Relative abundance of genera level

**Figure 6:**
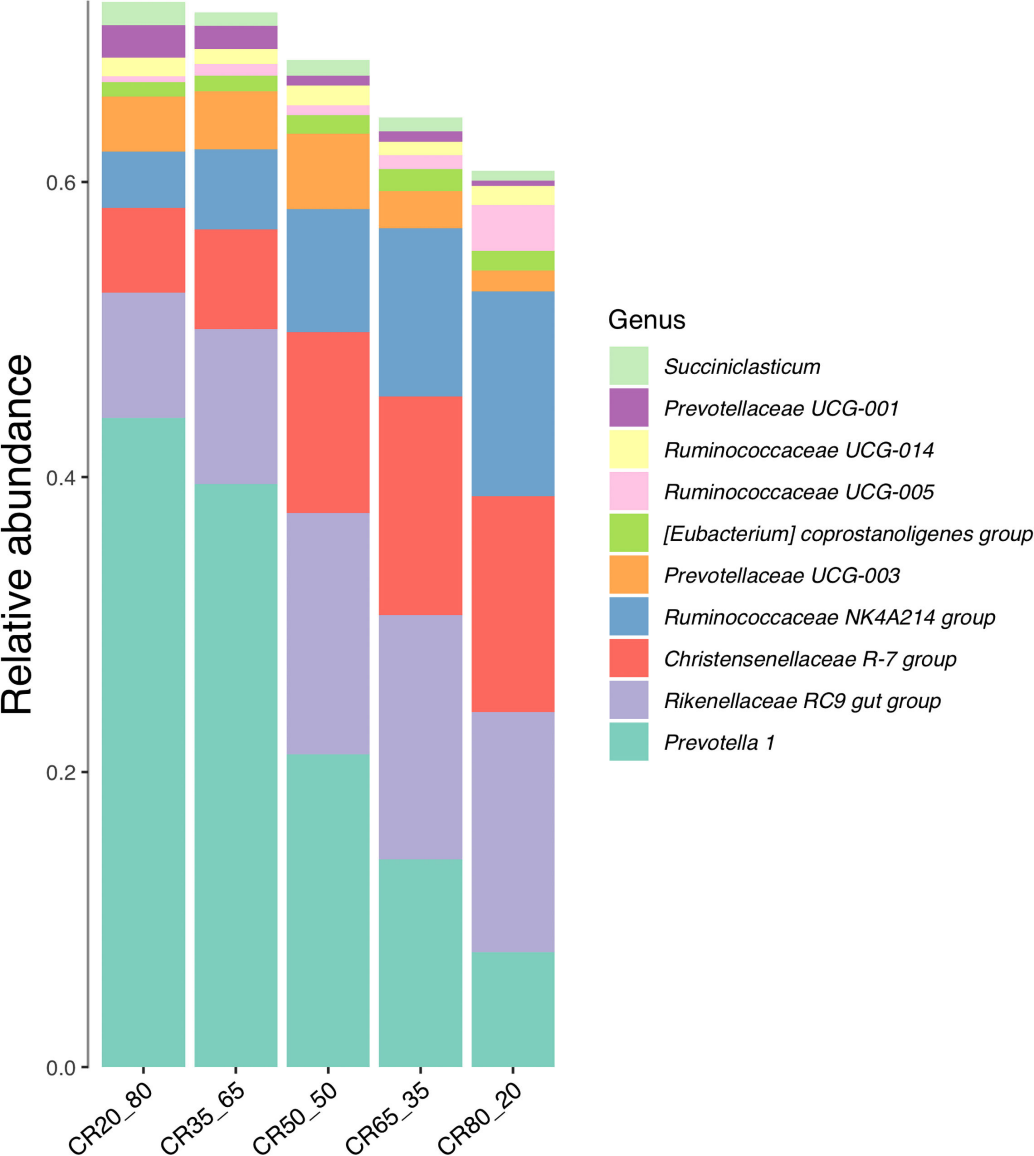
Composition of relative abundance of genera level in rumen fluid under different concentrate to roughage ratios.

**Figure 6** is the relative abundance of the top 10 genera in the five groups. As you can see from the picture. The top 10 bacteria in relative abundance were *Prevotella 1*, *Rikenellaceae RC9 gut Group*, *Christensenellaceae R-7 Group*, *Ruminococcaceae NK4A214 Group*, *Prevotellaceae UCG-003*, *Prevotellaceae UCG-001*, *Treponema 2*, *g_Succiniclasticum*, *g_Ruminococcaceae UCG-014*, *Coprostanoligenes* group at the level of bacteria genera.

The relative abundance of *Prevotella 1* ranged from 7.89% to 44.15%. 20:80 group was 44.15%, 35:65 group was 39.37%, 50:50 group was 21.27%, 65:35 group was 14%, 80:20 group was 7.89%. The relative abundance of *Prevotella 1* in the experimental group showed a trend of decreasing with the increase of the concentrate to roughage ratio.

The relative abundance of the *Rikenellaceae RC9* gut group ranged from 8.74% to 16.62%. The highest was 16.62% in the 65:35 group, followed by 16.49% in the 80:20 group, 16.36% in the 50:50 group, 10.71% in the 35:65 group and 8.74% in the 20:80 group. In this study, the relative abundance of *Rikenellaceae RC9 Gut Group* in the experimental group showed a trend of first increasing and then decreasing with the increase of the concentrate to roughage ratio.

The relative abundance of the *Christensenellaceae R-7* group ranges from 5.99% to 14.88%. The results showed that the 80:20, 65:35, and 50:50 groups were 14.88%, 14.64%, and 12.49% respectively. The second was 6.61% in the 35:65 group and 5.99% in the 20:80 group. The relative abundance of the *Christensenellaceae R-7* group increased with the increase in the concentrate to roughage ratio.

The abundance of *Prevotellaceae UCG-003* ranged from 1.42% to 5.1%, with the highest at 5.1% in the 50:50 group and the lowest at 1.42% in the 80:20 group. The abundance of *Prevotellaceae UCG-001* ranged from 0.32% to 2.22%, and the highest was 2.22% in the 20:80 groups. The lowest was 0.32% in the 80:20 group.

### 3.10 Correlation between rumen microbiota and dietary concentrate to roughage ratio and different microbiota

**Figure 7:**
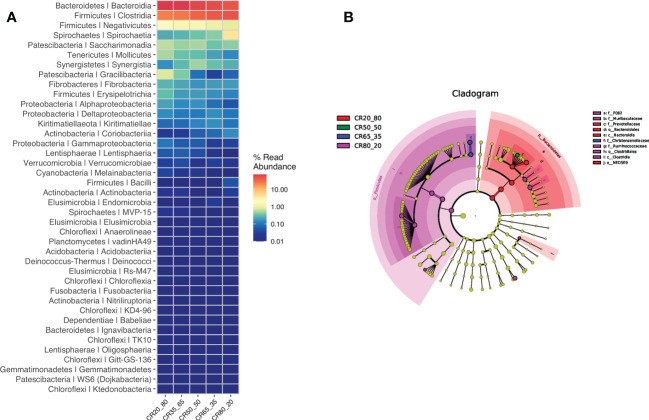
**(A)** shows the major bacteria detected. **(B)** shows the different rumen bacterial microbiotas.

The test detected bacteria are mainly *Bacteroidetes/Prevotellaceae, Firmicutes/Ruminococcaceae, Bacteroidetes/Rikenellaceae, Firmicutes/Christensenellceae, Bacteroidetes/F082, Firmicutes/Lachnospiraceae, Bacleroidetes/Bacteroidales UCG - 001*. The detection results are presented in **Figure 7A**.

Ten different rumen bacterial microbiotas were found in the four diets of 20:80, 50:50, 65:35, and 80:20. These differences are *f_F082*, *f_Murlbaculaceae*, *f_Prevotellaceae*, *o_Bacteroldales*, *C_Bacteroldla, f_Chrlstensenellaceae and f_Rumlnococcaceae*, *o_Clostrldlales, C_Clostrldla*, *o_NED5E9*. The different rumen bacterial microbiotas in **Figure 7B**.

### 3.11 Rumen bacteria changes with dietary nutrient composition

#### 3.11.1 Influence of ratio of raw material to core microbiota

**Figure 8:**
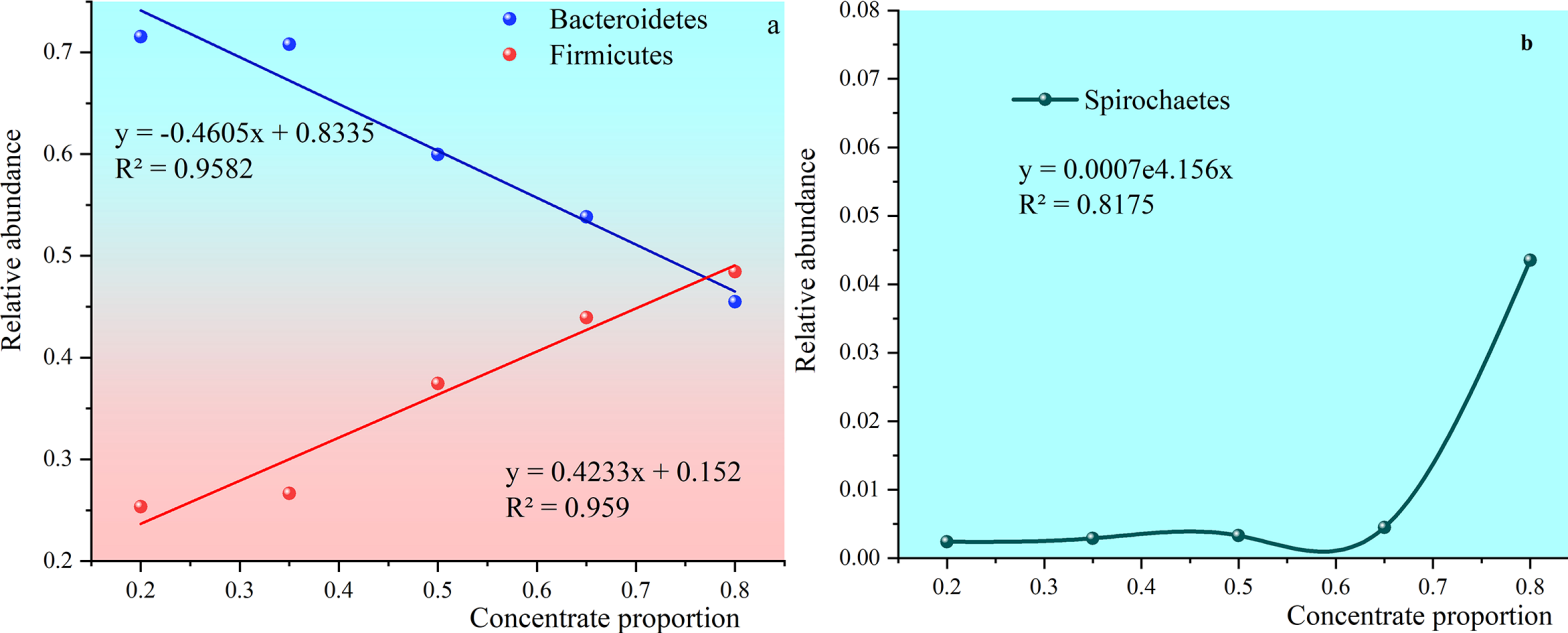
Trends in the abundance of Bacteroidetes, Firmicutes, and Spirochaetes with the increasing percentage of concentrate. **(A)** shows trends in the abundance of Bacteroidetes and Firmicutes with the increasing percentage of concentrate. **(B)** shows trends in the abundance of Spirochaetes with the increasing percentage of concentrate.


**Figure 8** shows that the relative abundance of *Bacteroidetes*, and *Firmicutes* exceeds 80%. The relative abundance of *Bacteroidetes* in all groups except the 80:20 group was higher than that in *Firmicutes*. The relative abundance of *Bacteroidetes* gradually decreased with the increase of concentrate to roughage ratio. The relative abundance of *Firmicutes* gradually increased with the increase of the concentrate to roughage ratio.

$$\left(\text{The relative abundance of Bacteroidetes}\right)\;=\;-0.4605\times \;\left(\text{percentage of concentrate}\right)\;+0.8335,{\text{ R}}^{2}=0.9582.$$

$$\left(\text{The relative abundance of Firmicutes}\right)\;=\;0.4233\times \;\left(\text{percentage of concentrate}\right)\;+0.152,{\text{ R}}^{2}\;=\;0.959.$$

The relative abundance of *Firmicutes* in group 80:20 was 48.45% and higher than *Bacteroidetes*. The two trend lines of relative abundance crossed. After solving the trend line simulation equations, it was concluded that the percentage of the crossing point in the concentrate supplement was 75.62%.

*Spirochaetes* can efficiently degrade pectin and phosphate esters and synthesize volatile fatty acids from fermentable carbohydrates (Nguyen et al., 2012). The relative abundance was 0.24%, 0.29%, 0.33%, 0.45% and 4.35% as the ratio of concentrate to roughage increased.


$$\left(\text{The relative abundance of Spirochaetes}\right)\;=\;0.0007{\text{e}}^{4.156(\text{percentage of concentrate})},{\text{ R}}^{2\;=}0.8175.$$

The relative abundance of the rumen microbiota was staggered with the change in the dietary nutrient composition. In the first case, the relative abundance of one microbiota decreased and the relative abundance of the other microbiota increased with the change in diet.

The relative abundance of these two groups of rumen bacteria dominated the core microbiota. The alternation of their relative abundance means that the original fermentation type may be disturbed or even broken. The relative abundance of *Bacteroidetes* was higher when the concentrate to roughage ratio was below 65:35. But with the increase of concentrate to roughage ratio, the relative abundance of *Bacteroidetes* decreased, and the relative abundance of *Firmicutes* increased. The relative abundance of *Firmicutes* exceeded that of *Bacteroidetes* before the ratio of concentrate to rougher was close to 80:20. The relative abundance of the two is equal when the ratio of concentrate to roughage is about 75.26%.

The relative abundance alternated with that of *Patescibacteria* and *Spirochaetes*, but their relative abundance was only 0.2% ~ 1.3%. With the increase of concentrate to roughage ratio, the relative abundance of *Spirochaetes* decreased and that of *Patescibacteria* increased. The relative abundance of the two is equal when the proportion of concentrate is 65%.

In the second case, the relative abundance of multiple bacterial communities increased or decreased with diet, but the trend lines intersected because of the different slopes of the increase and decrease. For example, the *g_Lachnospiraceae AC2044 group* and *o_Mollicutes RF39 | f_ | g* relative abundance decreased with higher protein content. The relative abundance of the two is equal when the ratio of concentrate is 65%.

In the third case, the relative abundance trend line of the microbiota is curved and intersects with the trend lines of the microbiota. For example, the relative abundance of the *Symtrophs* is a parabola with an opening downward, and the relative abundance of the *Fibrinobacteria* rises slowly in a straight line as the ratio of concentrate to roughage increases.

**Figure 9:**
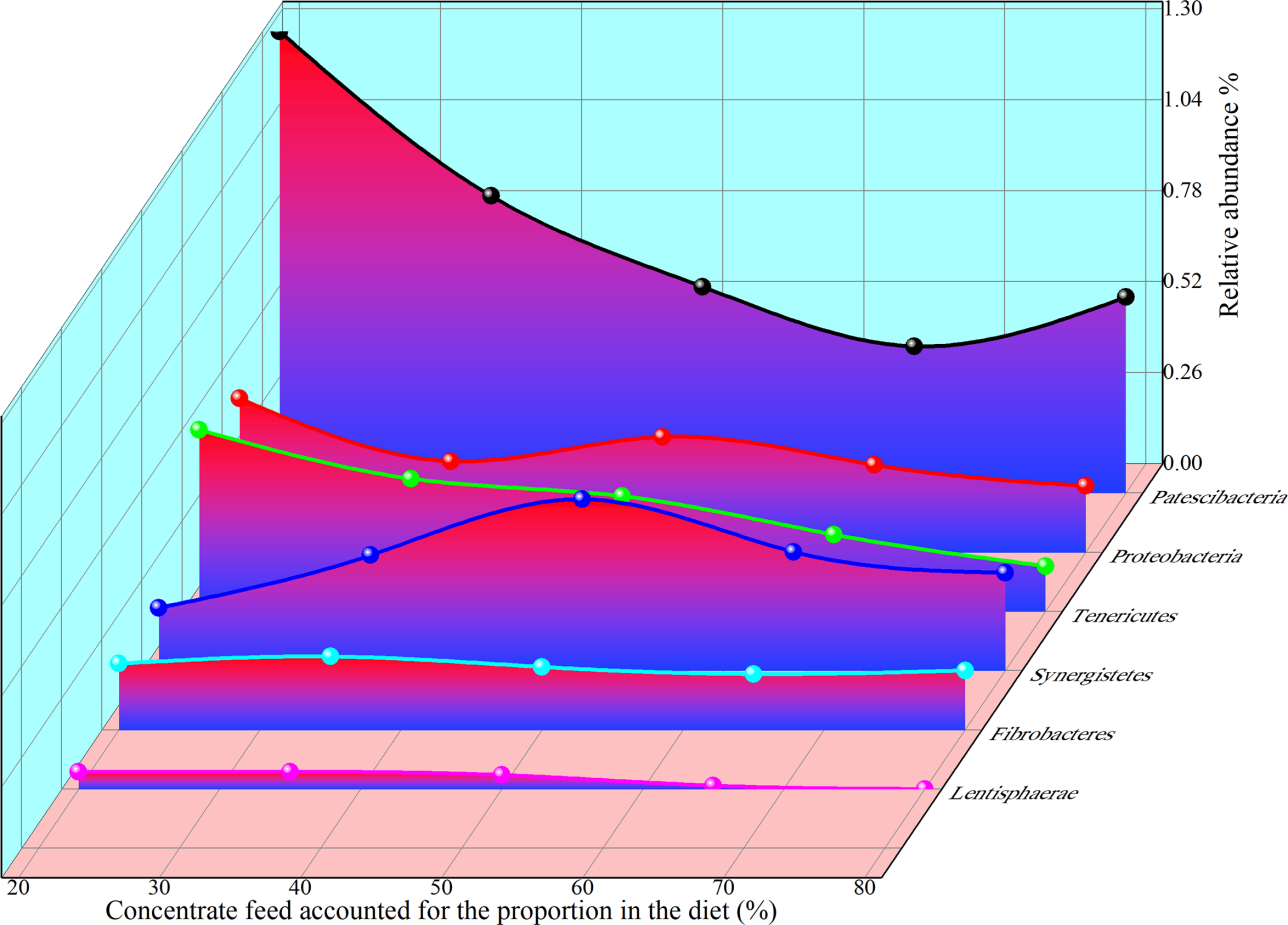
Relative abundance and concentrate fraction (%) of Phylum.

As shown in the **Figure 9**, the relative abundance of *Patescibacteria*, *Proteobacteria*, *Kiritimatiellaeota*, *Tenericutes*, *Lentisphaerae*, *Synergistetes*, and *Fibrobacteres* showed regular changes with the increase of concentrate to roughage ratio. The simulation equation as shown in **Suppl Table 1**, was obtained based on the data of relative abundance and concentrate proportion. The simulation equations for the relative abundance of these microbiota as a function of the concentrate proportion are presented in **Suppl Table 1**.

**Figure 10:**
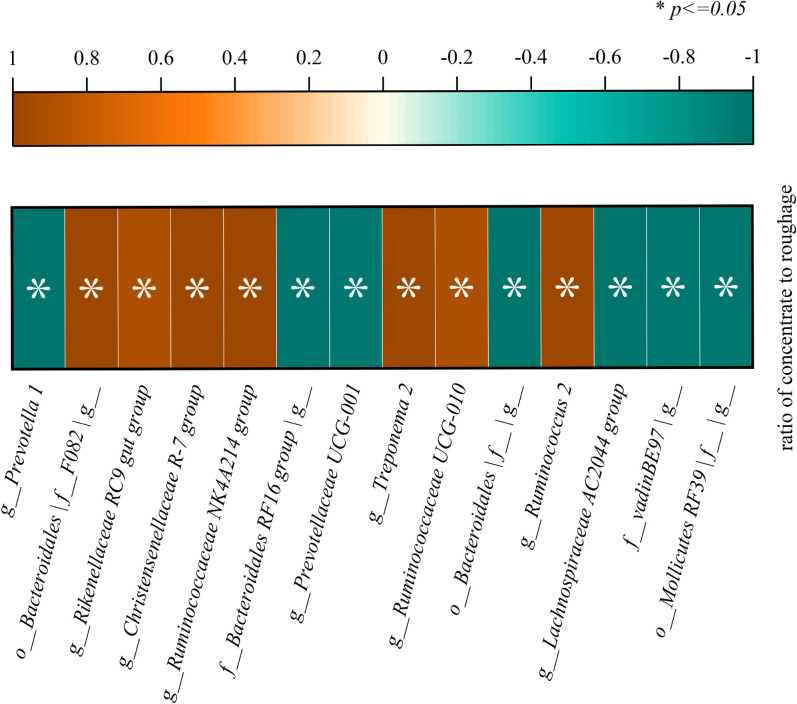
There was a significant correlation between bacterial genus level and concentrate to roughage ratio(*p<0.05*).

The significant correlation between bacterial genus level and concentrate to roughage ratio is shown in **Figure 10**. A significant correlation was found with the ratio of concentrate to roughage for the microbial genera *g_Prevotella 1*, *f_Bacteroidales RF16 group|g*, *g_Prevotellaceae UCG-001*, *g_Lachnospiraceae AC2044 group*, *o_Bacteroidales|f_|g*, *o_Mollicutes RF39|f_|g*, *g_Ruminococcaceae UCG-010*, *f_vadinBE97|g*, *g_Rikenellaceae RC9 gut group*, *o_Bacteroidales |f_F082|g*, *g_Christensenellaceae R-7 group*, *g_Ruminococcaceae NK4A214 group*, *g_Ruminococcus 2*, *g_Treponema 2* (*P*<0.05). The simulation equations for relative abundance and concentrate fraction of genera as shown in **Suppl Table 2**.

## 4 Discussion

### 4.1 Feed intake and daily gain

Higher feed intake was found in the 50:50 group and the 35:65 group. This may be because these two groups of concentrate have an appropriate proportion, good palatability, and contain an appropriate amount of fiber used for rumen microbial fermentation. Experimental animals like to eat such diets and it will not cause abnormal rumen fermentation. In this study, the proportion of concentrate supplements reached the highest feed intake and daily gain at 50:50 group. The results showed that the daily gain, total volatile fatty acids, and apparent digestibility were all in the 50:50 group, and the 35:65 group were all higher.

Hanlon et al. found that feed intake on a high-starch diet was 21.4% higher than that on a low-starch diet in a Holstein dairy feeding trial (Raffrenato et al., 2018). This experiment showed that the highest feed intake was 50:50 group (starch content was 26.06%), but the total feed intake and daily gain began to decrease when the concentrate to roughage ratio continued to rise. According to the results of the experiment, it is speculated that high starch will lead to a decrease in buffalo feed intake. Dietary energy concentration is an important factor affecting animal feed intake. Animals eat for energy, and feed intake may decrease significantly with the increase in feed energy level. Changing energy concentration is used as a way to regulate feed intake in feed preparation. When ruminants lack CF in their diets, the homeostasis mechanisms of chyme flow, gas excretion, and pH regulation are disrupted, and the animals are severely affected (Russell and Rychlik, 2001). Some of the above conditions may have occurred in the 80:20 group in this experiment.

**Figure 11:**
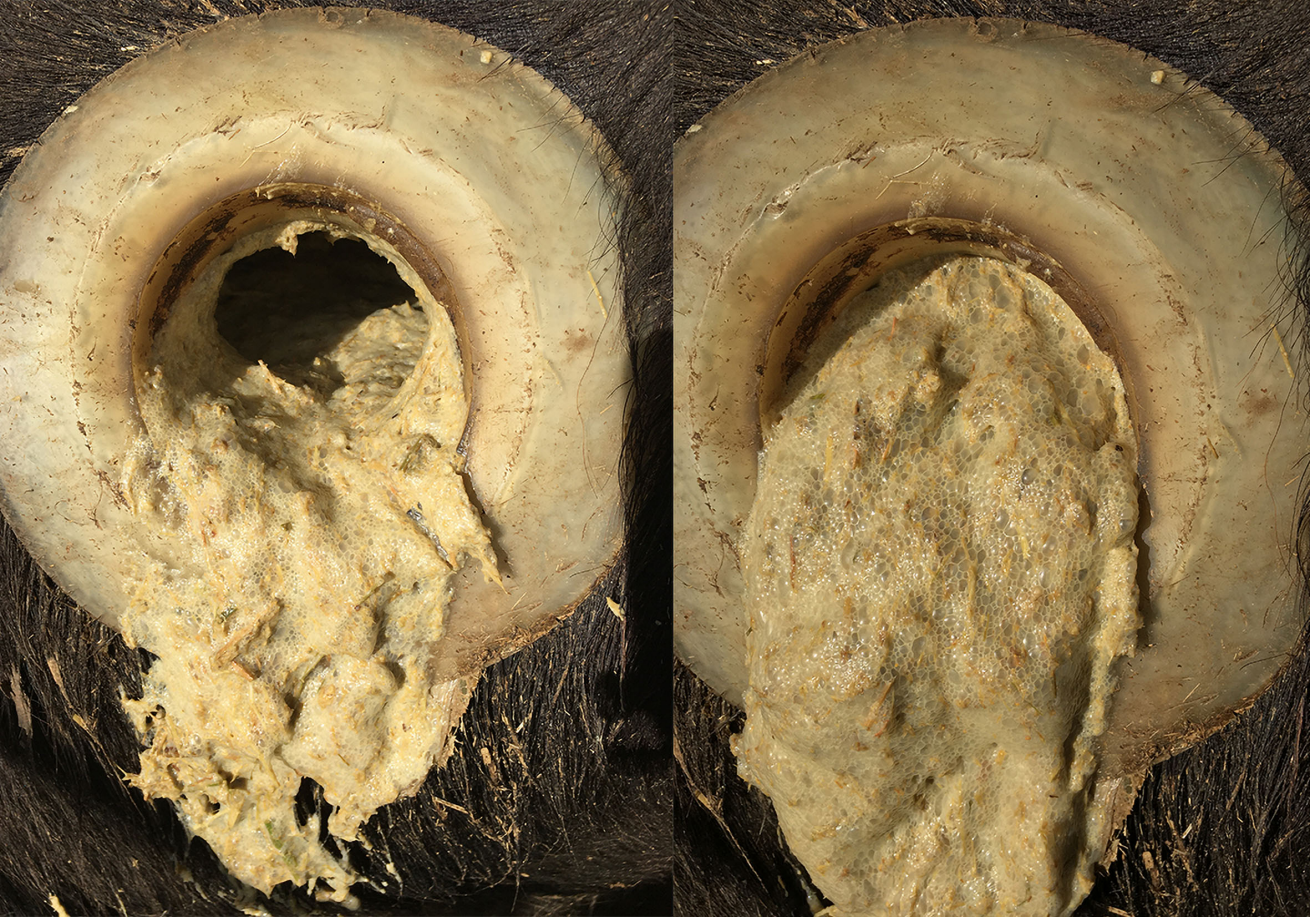
Foams were found in the 80:20 group.

The presence of large amounts of foam in rumen contents was observed in the 80:20 groups (The picture lacks comparisons of all test groups and is not suitable for the results section). It was found that pH was the lowest, feed intake decreased, and total volatile fatty acid concentration decreased in the 80:20 group. This may be due to abnormal rumen fermentation due to the extremely high concentration. The 80:20 group was the only experimental group that showed weight loss among the 5 experimental groups in this experiment. The researchers found that the foams were thick and resistant to rupture after observing the 80:20 groups of foams produced in the rumen. These foams rise with the rumen fluid level and adhere to the entire rumen wall, and when the rumen contracts (a large amount of foam pours out when the fistula was opened). As shown in **Figure 11**. These foams do not burst in time until the next rumen contraction, during the rumen stretch. Under normal conditions, there is no foam in the rumen fluid. These foams seriously affect the absorption of nutrients by the rumen.

### 4.2 The influence of different diets on pH

Rumen fluid pH below 5.6 is considered subacute rumen acidosis, while pH below 5.0 is considered acute acidosis, according to existing studies (Russell and Rychlik, 2001). All groups had pH between 6.0 and 7.0 and the criteria for subacute rumen acidosis did not occur in this trial. Some researchers also pointed out that although rumen pH value is the most commonly used indicator of rumen acidosis, due to differences and other reasons, there is no complete consensus on the definition of rumen acidosis pH value at present. Furthermore, rumen pH cannot fully explain all symptoms of rumen acidosis (Huber, 1976). Faleiro et al., 2011 showed that rumen fluid pH per se was not the cause of reduced feed intake (Faleiro et al., 2011). A decrease or abnormal change in daily feed intake is considered one of the symptoms of subclinical acidosis.

It is therefore likely that subacute ruminal acidosis exists in 80:20 groups. The inferred reasons were decreased feed intake, significantly decreased body weight and abnormal rumen fermentation in 80:20 group. Many factors can alter rumen ecology, among which it has been confirmed that a high grain and low fiber diet significantly reduces rumen pH value (Ampapon et al., 2019). The 80:20 group of anastomotic ruminants were fed diets deficient in fiber, which resulted in some symptoms of mixed movement, less belching and rumination, reduced saliva outflow, accumulation of fermented acid, and decreased rumen pH (Russell and Rychlik, 2001).

Previous studies have shown that plant nutrients play an important role in improving rumen fermentation as an effective chemical buffer. The stability of rumen pH within a certain range is the main factor to form the optimal rumen ecological environment. Especially when pH is lower than 6.0, microorganisms and the fermentation process will be seriously affected (Ampapon et al., 2019). Increasing the level of rapidly fermented starch in the diet will increase the rumen VFA production, which will exceed its absorption and buffering capacity, leading to a decrease in rumen pH (Hanlon et al., 2020). In this experiment, the pH value increased with the proportion of roughage, which proved this view to some extent.

### 4.3 Effects of different diets on ammonia-nitrogen concentration in the rumen

The main source of ammonia nitrogen (NH_3_-N) in the rumen was the degradation of nitrogen substances in the diet. High or low concentrations of NH_3_-N directly affect the growth and reproduction of microorganisms, as it is a precursor for the synthesis of MCP by rumen microorganisms (Karimizadeh et al., 2017). Changes in the NH_3_-N concentration of rumen fluid occur with changes in the protein content of the diet. The ability of rumen microorganisms to break down cellulose decreases as rumen NH_3_-N concentration decreases when protein is in short supply, resulting in rumen microorganisms being restricted in growth due to insufficient nitrogen sources. As a result, the digestion and absorption of carbohydrates are insufficient to maintain the body’s demand, ultimately resulting in reduced animal performance (Huhtanen et al., 2015).

The minimum NH_3_-N concentration for rumen microbial survival is 2~5 mg/dL, and rumen NH_3_-N provides the main nitrogen source for fiber-degrading bacteria (Huhtanen et al., 2015; Karimizadeh et al., 2017). The concentrations of NH_3_-N detected in this trial ranged from 9.6 to 34 mg/dL, which is above the minimum limit. The release rate of NH_3_-N may be higher than the utilization rate of NH_3_-N by rumen microorganisms under the condition of high protein content in the diet. Combined with dietary CP content and apparent digestibility data, it is not difficult to find that dietary CP content in 80:20 groups and 65:35 groups was higher than those in 50:50 groups, and rumen NH_3_-N concentration in 80:20 groups and 65:35 groups was higher than those in 50:50 groups. But the CPD was 50:50 higher than 80:20 and 65:35.

The researchers reported that the optimum concentration of rumen NH_3_-N should be 5 to 27.5 mg/dL (Huhtanen et al., 2015; Karimizadeh et al., 2017; Antunes et al., 2019; Gruninger et al., 2019; Ciriaco et al., 2021). The highest concentration of NH_3_-N in this trial was in the 80:20 group at 31.00 mg/dL, which also had the highest protein content. The test confirmed a positive correlation between the protein content of the diet and the NH_3_-N concentration. However, the results of this experiment do not indicate that higher protein content is better in ruminant diets. Body weight decreased significantly and subacute rumen acidosis were observed in the 80:20 group with high protein content.

### 4.4 Effects of different diets on rumen volatile fatty acids

Volatile fatty acids (VFA) are produced by the degradation of carbohydrates in the ruminant diet. VFA is the primary source for the maintenance and growth of rumen microorganisms. The content and proportion of all kinds of VFA influence the growth of animals. Meanwhile, the concentration of VFA reflects the degree of carbohydrate degradation. The higher the proportion of concentrate supplement in daily diet, the higher the propionic acid content and the lower the ratio of acetic acid to propionic acid produced by rumen fermentation (Russell and Rychlik, 2001).

The variation trend of the total VFA, acetic acid, and propionic acid concentrations was consistent in this experiment. The concentration of acetic acid was higher than that of propionic acid throughout the experiment. The difference between the highest and lowest concentrations of acetic acid and propionic acid was nearly four-fold. In 2020, Hanlon et al. (2020) obtained that the total VFA in the high-starch group was significantly higher than that in the low-starch group in the feeding experiment of Holstein dairy cows using high-starch and low-starch diets, which was consistent with this experiment. When concentrate supplementation increased, the concentrations of acetic acid, propionic acid, and butyric acid in the rumen increased simultaneously, which led to the increase of total volatile fatty acids.

According to the data of 20%, 40%, 60%, and 80% of concentrate supplements, Zhang et al. (2017) experiment in 2017 showed that acetic acid was the volatile fatty acid with the highest content in all test groups, which was consistent with this test. Zhang et al. pointed out that the group with the highest acetic acid content was the group with the highest roughage proportion in the experiment. However, the highest concentration of acetic acid in this experiment was in the 35:65 group.

### 4.5 Effects of different diets on rumen bacterial phylum levels

#### 4.5.1 Effects of different diets on rumen bacterial phylum levels

*Firmicutes* and *Bacteroidetes* are the core rumen microbiome, both of which play a crucial role in rumen fermentation (Weimer, 2015). In this study, the sum of the relative abundance of *Bacteroidetes* and *Firmicutes* exceeded 80%, which was consistent with previous studies. The experimental results of Jami, Zhang, and Pitta et al. showed that the relative abundance of *Bacteroidetes* in the rumen fluid of ruminant animal fed with different diets was higher than that of *Firmicutes* (Jami and Mizrahi, 2012; Zhang et al., 2014; Pitta et al., 2014). In this experiment, however, the relative abundance of *Firmicutes* was higher than that of *Bacteroidetes* in the 80:20 group. Combined with previous studies, it was concluded that the dominant bacteria in rumen microbes were *Bacteroidetes* followed by *Firmicutes* when buffalo diets were roughage-based (Pitta et al., 2013). Experimental data showed that the relative abundance of *Bacteroidetes* decreased with the increase of the concentrate to roughage ratio. The relative abundance of *Firmicutes* increased with the increase of the concentrate to roughage ratio. In this trial, when the proportion of concentrate feed exceeded 75.62%, the first dominant bacteria in the rumen microbes of Dehong three-way hybrid buffalo were *Firmicutes* instead of *Bacteroidetes*.

*Bacteroidetes* are an essential component of the rumen microbiota, and their main function is the fermentation of starch, pectin, and other carbohydrates. The relative abundance of *Bacteroidetes* decreased with the increase of concentrate supplements. There are two possible reasons for this. First, Part *Bacteroidete*s provide nutrients to the body by degrading cellulose, pectin, and complex carbohydrates (Won et al., 2020). The increase in the concentrate to roughage ratio leads to a decrease in fiber content and complex carbohydrates, which indicates the fermentation substrate of *Bacteroidetes*. This results in a decrease in the relative abundance of *Bacteroides* in this part. Second: cellulose decomposing bacteria in the rumen can produce fibrous hydrolase for decomposing cellulose. Ruminants can digest cellulose efficiently mainly because of the action of such bacteria. Cellulolytic bacteria are sensitive to pH changes. The growth and reproduction of cellulose-decomposing bacteria will be inhibited, resulting in a decrease in the roughage digestibility of ruminants when the pH value is lower than 6.2 (Pitta et al., 2010; Rosenberg, 2014). Combined with the experimental data of 35:65, 50:50, 65:35, and 80:20 groups, it was found that with the increase of concentrate to roughage ratio, pH value, cellulose digestibility, and food intake decreased. The decrease in pH, cellulose digestibility and food intake is consistent with the above theory.

The data from this trial showed that the relative abundance of *Firmicutes* increased with increasing concentrate to roughage ratio. *Firmicutes* contain a large number of fiber-degrading bacteria. However, these fiber-degrading bacteria can also use starch and protein as substrates. For example, *Butyrivibrio fibrisolven* can quickly and thoroughly degrade cellulose. *Butyrivibrio fibrisolven* has a wide range of fermentable substrates and can grow on starch, pectin polysaccharides, and other non-starch polysaccharides. Meanwhile, *Butyrivibrio fibrisolven* also has protease degradation activity and is one of the main protein-degrading bacteria in the rumen. The largest genus of bacteria under the Firmicutes is Clostridium, which can degrade nutrients such as fiber, starch, protein, and titin. In addition, the important starch degrading bacteria in the rumen - Ruminococcus also belongs to *Firmicutes*. The *Firmicutes* contain abundant bacteria that can degrade concentrate supplements, causing their relative abundance to increase as the concentrate to roughage ratio rises.

Studies have shown that *spirochetes* can effectively degrade pectin and phosphate esters and synthesize volatile fatty acids from fermentable carbohydrates to provide energy for ruminants (Hess et al., 2011). The results showed that the relative abundance of spirochaetes increased with the increase in the ratio of concentrate to roughage. Stewart CS et al. pointed out that spirochetes had stronger fermentation and conversion capacity for carbohydrates and different types of fatty acids than *Prevorella*. Other bacteria with this ability are *Succinvibrio* and *Tuberococcus* (Zened et al., 2013). The change trends of the concentrate to roughage ratio, starch content, and NFC digestibility, were consistent with the relative abundance of the spirochete. The relative abundances of other bacteria phylum showed regular changes with the dietary concentration to roughage ratio.

#### 4.5.2 The relative abundance of microbiota staggered with the change in dietary nutrient composition

*Prevotella* plays a role in protein degradation and fermentation, and p*revotella* with protease activity is prevalent in the rumen (Rosenberg, 2014). However, the relative abundance of *Prevotella* in this experiment did not increase with the increase of dietary protein content but decreased. According to the experimental results, the relative abundance of *Prevotella* decreased with the increase of dietary starch content, relative abundance increased with the increase of ADF and NDF. These results indicated that the relative abundance of *Prevotella* decreased with the increase of the concentrate to roughage ratio. Alexandre B. et al. found in the experiment comparing forage and total mixed ratio that the relative abundance of *Prevoella* was higher in the roughage group than in the total mixed ratio group (Menezes et al., 2011). Khafipour et al. showed in subacute rumen acidosis induced test of cattle that *prevorella* abundance decreased under subacute rumen acidosis (Khafipour et al., 2009). Golde et al. also showed that the relative abundance of *Prevotella* was higher in the control group and lower in the experimental group with higher nutritional levels. The acidosis characteristic value of *Prevotella* was higher than that of other bacteria (Golder et al., 2014). The relative abundance of *Prevotella* decreased with the increase of the concentrate to roughage ratio, which may be due to the decrease of rumen pH value, which inhibited the growth of *Prevotella*.

Similarly, the relative abundance of the three genera *g_Ruminococcaceae NK4A214 group*, *g_Ruminococcaceae UCG-010*, and *g_Ruminococcus 2* increased with the increase of dietary protein content. The relative abundance of the three genera decreases with the increase of ADF and NDF content. The three rumen cocci mentioned above all belong to *Firmicutes*, which also explains why the relative abundance of *Firmicutes* increases with the increase of concentrate to roughage ratio. The relative abundance of *Ruminococcaceae* was higher in the experimental group with a higher proportion of concentrate, which was consistent with the results of Alexandre et al. (Menezes et al., 2011).

A certain amount of fiber is required in the ruminant diet to stimulate chewing activity, salivary buffer supply, rumen motility, and mixing, and to maintain the proper functioning of the rumen ecosystem (Humer et al., 2018). The relative abundance of *Fibrobacillus* decreased with the increase of the concentrate to roughage ratio. It was found that there was a clear relationship among fiber content, fiber digestibility, and relative abundance of *Fibrobacillus* (Weimer, 2015). Cellulose is not a limiting factor for the growth of microorganisms, when the amount of potentially digestible cellulose in the rumen is much higher than the digestible amount during the whole digestion time. At this time, the amount of cellulose as the substrate for fermentation is excessive (Weimer, 2015). The first case: high content of roughage in the diet. Although there is a change in cellulose content but little impact on microorganisms. In the case of concentrate gradually increasing, roughage gradually reduced. There is a possibility of insufficient biodegradable cellulose as substrate, and then the change of cellulose content gradually manifests its influence on microorganisms. The digestibility of fiber begins to decrease, and the relative abundance of *Fibromyta* begins to decrease when the fiber content reaches a certain height. According to the data of this experiment, the relative abundance of *Fibromyta*, ADFD, and NDFD reached the highest when the concentrate to roughage ratio was 35:65.

### 4.6 The change of relative abundance of bacterial genus level with dietary nutrient composition

Similarly, abundance trend lines at the genus level cross over as dietary nutrient composition changes. The relative abundance of *g_Prevotella 1* decreases with increasing concentrate to coarse ratio. The *o_Bacteroidales |f_F082| g_*, *g_Rikenellaceae RC9 gut group*, *g_Christensenellaceae R-7 group*, *g_Ruminococcaceae NK4A214 group* Relative abundance increased with increasing concentrate to coarse ratios. The relative abundance of *Prevotella 1* was equal to the four bacterial genera when the proportion of dietary concentrate proportion was about 65%. The *g_Ruminococcaceae UCG-010*, and *g_Ruminococcus f_Bacteroidales RF16 group| g_*, *g_Prevotellaceae* relative abundance of *UCG-001* cross the trend line when the proportion of dietary concentrate proportion was about 60%.

The relative abundance of rumen bacteria began to alternate when the percentage of concentration was more than 60%. The relative abundance of the two largest core phyla alternated when the percentage of concentration reached about 75%. The reason may be that the microbiota with lower relative abundance is less stable and more vulnerable to fragmentation, while the microbiota with higher relative abundance is more stable. However, when the ratio of the concentration reached about 75%, the stability of the rumen system reached the upper limit, resulting in the alternation of the abundance of rumen core microbiota.

This phenomenon of alternation of relative abundance of rumen bacteria cannot be a simple generalization by the change of relative abundance of rumen bacteria with different diets. This phenomenon means the change of rumen fermentation type, the destruction of the rumen environment, the disorder of the bacterial community, and even the embodiment of acidosis.

Combined with the rumen environmental data, the rumen pH value was close to the minimum value, NH_3_-N was close to the maximum value, and total VFA was close to the maximum value when the rumen concentration to forage ratio of the alternating changes of relative bacterial abundance. These results propose that the alternation of relative bacterial abundance can be used as an indicator of herd health under the premise of understanding the structure of the rumen microbiota.

### 4.7 It was hypothesized that the variation trends of rumen bacteria abundance at different levels were overlapping.

The definition of subacute rumen acidosis is unclear. Rumen pH is defective as an indicator of acute rumen acidosis, because dietary differences, variety differences, and even individual health status can affect rumen pH (Lam et al., 2017). The relative abundance value of rumen microorganisms is a ratio, excluding the interference of absolute number, which can more objectively reflect the rumen fermentation state compared with pH value.

The alternating points of their relative abundance were used as the early warning points of subacute rumen acidosis after selecting several representative microorganisms from numerous rumen microorganisms. For example, the relative abundance of *Prevotella 1* was equal to the four bacterial genera when the proportion of dietary concentrate proportion was about 65%. A significant alternation point of relative abundance is used as an alarm point, such as the alternation point of *Bacteroidetes* and *Firmicutes* when the concentrate proportion was about 75%. In this way, subacute rumen acidosis/rumen acidosis can be accurately understood for each breed and even for each individual. The results of this study suggest that a new definition of subacute rumen acidosis/rumen acidosis is based on the relative abundance variation of rumen microbiota.

## 5 Conclusion

1 In this experiment, the daily gain and DMI (G/kgW0.75) reached the highest when concentrate accounted for 50%, but the apparent digestibility of ADF, NDF, NDICP, and ADICP reached the highest when concentrate accounted for 35%. The weight loss of the experimental animals when the concentration level reaches 80%.

2 Rumen pH decreased and rumen ammonia nitrogen increased with the increase of concentrate proportion. Rumen pH decreased linearly with the increase of dietary starch content and NFC content. Rumen NH_3_-N concentration increased linearly with the increase of dietary protein content. Rumen total volatile fatty acid concentration (mmol/L) decreased linearly with the increase of dietary NDF, and linearly increased with the increase of NFC and CP, and the acetic acid proportion increased linearly with the increase of NDF.

3 Rumen bacteria in diets with different concentrate to roughage ratios were not only different, but the relative abundance of each bacterial community changed regularly with the change of concentrate to roughage ratio. The relative abundance of rumen core microbiota of *Bacteroidetes* and *Firmicutes* alternated with the change of concentrate ratio. This proposes that the relative abundance of rumen microbiota or the ratio of several rumen microorganisms can be used to specify and even measure rumen health, even rumen subacute rumen acidosis/rumen acidosis.

## Data availability statement

The data presented in the study are deposited in the NCBI repository, accession number PRJNA888478.

## Ethics statement

The animal study was reviewed and approved by Committee of the Yunnan Agricultural University (Contract 2007-0081). Written informed consent was obtained from the owners for the participation of their animals in this study.

## Author contributions

Experimental design guide, HM, QL, Animal feed: RW, SCH, DW, SYH, TG, TC, XF; The data processing: RW, SCH, The sample testing: RW, SCH, DW, SYH, TG, writing—original draft preparation: RW, DH, supervision: HP, CC, MH, LJ, DS, XH. All authors have read and agreed to the published version of the manuscript.
